# Structural chromosome aberrations cause swelling of the nucleus

**DOI:** 10.1186/s41021-016-0047-7

**Published:** 2016-10-01

**Authors:** Kenji Takeshita, Hiroaki I. Ogawa, Toshinari Maeda

**Affiliations:** 1 Department of Biological Functions Engineering, Graduate School of Life Science and Systems Engineering, Kyushu Institute of Technology, 2-4 Hibikino, Wakamatsu-ku, Fukuoka, 808-0196 Japan; 2 UBE Scientific Analysis Laboratory, Inc, 1978-5 Kogushi, Ube, Yamaguchi 755-8633 Japan

**Keywords:** Chromosomal aberration, Nuclear swelling, Clastogen, Genotoxicity, Aneugen, Carcinogen, Non-mutagen

## Abstract

**Background:**

Carcinogens are known to cause swelling of the mammalian cell nucleus. However, the mechanism of the swelling and its toxicological significance have not been fully elucidated. Since nuclear swelling (NS hereafter) has been frequently observed in chromosomal aberration (CA hereafter) tests (in vitro), the relationship between NS and CAs was investigated in this study.

**Results:**

In a short-term CA test using the fibroblast CHL cell line, the appearance of NS increased in a dose-dependent manner after exposure to six types of clastogens (mitomycin C, methyl methane sulfonate, 1-methyl-3-nitro-1-nitrosoguanidine, benzo[a]pyrene, cyclophosphamide monohydrate, and 9,10-dimethyl-2-benzanthracene), and a strong correlation was found between NS (%) and CAs (%) at each dosage. Therefore, we hypothesized that clastogens cause NS in cultured mammalian cells, since the mouse lymphoma L5178Y cell line is known to have a similar sensitivity to clastogens. Thus, we measured NS for 14 compounds (clastogens) that are known to induce structural CAs, 4 aneugens, and 12 non-mutagenes. Almost all clastogens caused NS of more than 5 %, which increased in a dose-dependent manner. Among the aneugens, colchicine, and diethylstilbestrol caused the same level of NS % as the clastogens, while carbendazim and trichlorfon caused a similar level of NS % as the clastogens only at higher levels of cytotoxicity. Almost all the non-mutagens caused less than 5 % NS.

**Conclusions:**

These results strongly suggest that NS is mainly caused by structural aberrations in the nucleus during interphase of the cell cycle.

## Background

Carcinogens cause swelling of the mammalian cell nucleus, a phenomenon first reported by Page [1]. Numerous studies have evaluated nuclear swelling (NS), including in cultured mammalian cells (e.g., HeLa S3 cells) by Agrelo [2]. Grant and Finch et al. proposed a screening test (nuclear enlargement assay) for identifying carcinogens based on NS [3, 4]. However, as described by Westmoreland et al., because most instances of NS are independent of abnormal enhancement of DNA synthesis [5], the mechanism of NS differs from those occurring in cellular senescence and cancerous cells [6, 7]. The mechanisms of NS and its toxicological significance remain unclear. Most carcinogens that cause NS are genotoxins, which cause DNA damage, chromosomal aberrations (CAs), and micronucleus formation. Because NS is often observed during the mammalian CA test (in vitro), we examined the relationship between NS and CAs. If NS is related to genotoxicity, it may be useful to measure NS in various cultured mammalian cell lines; we obtained additional information nearly simultaneously through additional microgram-scale sampling during the respective cell assays.

In this study, the correlation between NS and CAs was examined using CA tests for clastogens in the Chinese hamster lung CHL/IU cell line. NS measurements were carried out using compounds known to induce structural CAs in the mouse lymphoma L5178Y cell line. L5178Y is often used in mouse lymphoma L5178Y cell Tk (thymidine kinase) gene mutation assays. These assays can detect mutations in the Tk gene that result from both gene mutations and chromosome damage. It has also been reported that MMS, MNNG, 2AF, B (a) P, and DMBA cause chromosomal aberrations in L5178Y [8–11], and thus we hypothesized that L5178Y has a similar sensitivity to clastogens as the CHL [12, 13].

## Methods

### Cells

The CHL cell line was obtained from the Health Science Research Resources Bank (HSRRB, Osaka, Japan). The CHL cells were cultured in Eagle’s MEM supplemented with 10 % (v/v) heat-inactivated calf serum. Mouse lymphoma L5178Y TK^+/−^3.7.2c cells (L5178Y hereafter) were donated by the National Institute of Health Sciences (Tokyo, Japan). The L5178Y cells were cultured in RPMI1640 supplemented with 10 % (v/v) heat-inactivated horse serum and sodium pyruvate (200 μg/ml) in a humidified incubator at 37 °C with 5 % CO_2_.

### Chemicals

The chemicals used were chosen according to previous studies [14–21]. The tested compounds included 14 clastogens, 4 aneugens, and 12 non-mutagens (Table 1). These compounds were dissolved in dimethyl sulfoxide (DMSO) or physiological saline. The final concentration of the solvent to the culture medium was adjusted to 1 % (v/v) for each compound. The highest concentration for each compound was selected based on the minimum concentration that resulted in cytotoxicity. However, when a test compound showed no cytotoxicity, it was fixed to 500 μg/ml for clastogens and aneugens and to 2000 μg/ml for non-mutagens. The concentrations selected for analysis were in ratios of 2 or 4 to cover a wide concentration range (except for the ratio of √2 for cyclophosphamide).

**Table 1:** List of compounds tested

No.	Compound (Another name)	CAS No	Source	Solvent used
	Clastogens			
1	Mitomycine C (MMC)	50-07-7	Wako	Saline
2	Methyl methane sulfonate (MMS)	66-27-3	Sigma-Aldrich	Saline
3	1-Methyl-3-nitro-1-nitrosoguanidine (MNNG)	70-25-7	Wako	DMSO
4	Benzo[a]pyrene (B[a]P)	50-32-8	Wako	DMSO
5	Cyclophosphamide Monohydrate (CP)	6055-19-2	Wako	Saline
6	9,10-Dimethyl-1,2-benzanthracene (DMBA)	57-97-6	Wako	DMSO
7	Acetaminophen	103-90-2	Wako	DMSO
8	Sodium Azide	26628-22-8	Naca	Saline
9	2-(2-Furyl)-3-(5-nitro-2-furyl)-acrylamide (AF2)	3688-53-7	Wako	DMSO
10	4-Nitroquinoline 1-Oxide (4NQO)	56-57-5	Wako	DMSO
11	2-Acetamidofluorene	53-96-3	Wako	DMSO
12	1-Methyl-5H-pyrido[4,3-b]indole-3-amine・acetic acid (Trp-P-2 Acetate)1-Methyl-5H-pyrido[4,3-b]indole-3-amine・acetic acid (Trp-P-2 Acetate)	72254-58-1	Wako	DMSO
13	2-Aminoanthracene	613-13-8	Naca	DMSO
14	Diphenylamine	122-39-4	Wako	DMSO
	Aneugens			
15	Methyl 2-benzimidazole carbamate (Carbendazim)	10605-21-7	Wako	DMSO
16	Colchicine	64-86-8	Wako	DMSO
17	Diethylstilbestrol (DES)	56-53-1	Wako	DMSO
18	2,2,2-Trichloro-1-hydroxyethyl dimethyl phosphate (Trichlorfon)	52-68-6	Wako	DMSO
	Non-mutagens			
19	Toluene	108-88-3	Wako	DMSO
20	o-Chlorotoluene	95-49-8	Wako	DMSO
21	Salicylic acid	69-72-7	Wako	DMSO
22	DL-Tartaric acid	133-37-9	Wako	Saline
23	2,4,6-Trichloroaniline	634-93-5	Wako	DMSO
24	Diethyl phthalate	84-66-2	Wako	DMSO
25	L(+)-Ascorbic acid (Vitamin C)	50-81-7	Wako	Saline
26	β-Carotene	7235-40-7	TCI	DMSO
27	Methacrylamide	79-39-0	Wako	Saline
28	Cyclohexene	110-83-8	Wako	DMSO
29	Isopropylamine	75-31-0	Wako	Saline
30	dl-a-Tocopherol (Vitamin E)	10191-41-0	Wako	DMSO

Wako: Wako Pure Chemical Industries, Ltd

Naca: NACALAI TESQUE, INC

TCI: Tokyo Chemical Industry CO., LTD

### Metabolic activation system

To determine the influence of metabolic activation for a test compound, S9 mix was used. The S9 fraction was prepared from male Sprague–Dawley rats that were pretreated with the enzyme-inducing agents phenobarbital and 5, 6-benzoflabone, which were obtained from the Oriental Yeast Co. Ltd (Tokyo, Japan). In the experiment, S9 mix was added so that the S9 fraction concentrations were 5 % (v/v) for CHL and 2 % (v/v) for L5178Y cells in the final test medium. The S9 mix for CHL cells was prepared as follows: 2 ml of S9, 1.34 ml of 20 mM HEPES, 0.67 ml of 50 mM MgCl_2_, 0.67 ml of 330 mM KCl, 0.67 ml of 50 mM glucose-6-phosphate, 0.67 mM of 40 mM NADP(Na_2_), and 0.67 ml of purified water. The S9 mix for L5178Y cells was prepared as follows: 2 ml of S9, 1 ml of 0.18 g/ml glucose-6-phosphate, 1 ml of 25 mg/ml NADP, and 1 ml of 150 ml KCl.

### Treatment for CHL cells

Five milliliters of culture medium (4000 cells/ml) was added to a plastic Petri dish (60-mm diameter). After a 3-day incubation, the cells were exposed to the test compounds for 6 h. When appropriate, S9 mix (for metabolic activation) was also added to the medium. Furthermore, an 18-h recovery time was needed, and 2 h before cell sample collection, colcemid was added to the medium at a final concentration of 0.2 μg/ml in order to harvest the cells in the metaphase state. Trypsin was used to separate the cells from the Petri dishes, and the obtained cells were used to measure or estimate NS and CAs.

### Treatment for L5178Y cells

Cells (400 μl at a density of 5 × 10^5^ cells/ml) in the exponential cell growth phase were cultured in 1.5 ml micro-tubes, and the prescribed amounts of the test compounds were added, followed by the proper amount of 150 mM KCl (without metabolic activation) or S9 mix (with metabolic activation). After shaking gently at 37 °C in an incubator for 3 h, the cells were collected using centrifugation, and the supernatant was removed. One milliliter of fresh medium was added to the micro-tube, and the mixture was transferred to a 24-well plate. After a 21-h incubation, NS was measured and analyzed.

### Analyses of CAs

Cells were suspended and incubated in a 0.075 M KCl hypotonic solution for 15 min at 37 °C, and they were then fixed in ice-cold Carnoy solution (1:3 = acetic acid: ethanol). The fixed cell suspension was dropped on a glass slide, air-dried, and stained with Giemsa solution. A total of 100 cell images in metaphase were observed per dose with a microscope (Olympus BX50) at 600× magnification. When the number of metaphase cells was less than 100 because of cytotoxicity, all metaphase cells were counted per dose.

### Measurements of NS

Cell suspensions were fixed by adding formalin to a final concentration of 2 % (v/v), and the suspensions were then fluorescent stained by adding 50 μg/ml DAPI or 50 μg/ml Hoechst 33342 at a final concentration of 1 to 2 % (v/v) of the suspension. For CHL cells, the cell density was concentrated to 2 to 4 times, and the concentrated cell suspensions were placed between two glass plates with a 0.07-mm space using Sekisui microscopy plates (UR-137-S) obtained from Sekisui Chemical Co., Ltd. A total of 72 pictures (frames) of the cells per dose were taken with a fluorescence microscope (Olympus BX50FLA equipped with a 20× objective lens and a Nippon Roper chromosome inspection supporting system CIS-02) at a frame size of 646 μm × 483 μm. This was equivalent to taking pictures of 1.57 μl of the cell suspension. The resulting image data were analyzed using Image-Pro Plus image analysis software (ver. 5), and the number of cell nuclei was counted, and their area was measured. Using standard measures of nuclear size, NS was discriminated from other conditions.

### Cytotoxicity assessment

The following formula for cytotoxicity assessment was used:

Relative Increase in Cell Counts (RICC) [22–24]

$$ \mathrm{RICC}=\frac{\left(\mathrm{Increase}\ \mathrm{in}\ \mathrm{number}\ \mathrm{of}\ \mathrm{cells}\ \mathrm{in}\ \mathrm{treated}\ \mathrm{cultures}\ \left(\mathrm{final} - \mathrm{starting}\right)\right)}{\left(\mathrm{Increase}\ \mathrm{in}\ \mathrm{number}\ \mathrm{of}\ \mathrm{cells}\ \mathrm{in}\ \mathrm{control}\ \mathrm{cultures}\ \left(\mathrm{final}-\mathrm{starting}\right)\right)}\times 100 $$

### Statistical analysis

Fisher’s exact tests and Chi-squared tests were used to determine whether there were statistical differences between the negative control group and the treated group in the appearance of NS. For clastogen tests using CHL cells, the correlation coefficient (r^2^) was calculated to evaluate the correlation between NS (%) and CA (%) at each concentration. The significance level was set at *p* < 0.05 for all statistical analyses.

## Results

### Nuclear size

**Fig. 1:**
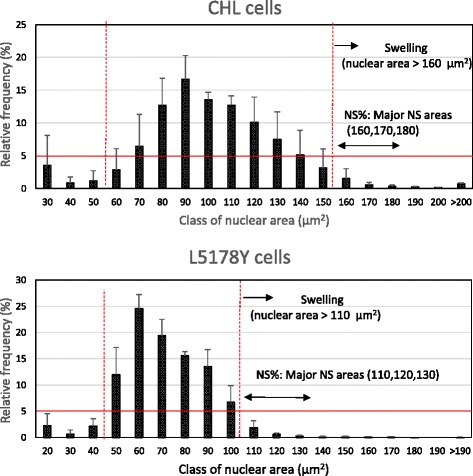
Histogram of cell nuclear areas for the negative controls. The ordinates (relative frequency) are expressed as the mean ± standard deviation (SD) values of six experiments. The error bars indicate the SD

As shown in Fig. 1, since the maximum value of the standard deviation (SD) was approximately 5 % for the frequency distribution of the cell nucleus area in the negative control group, a relative frequency (average + SD) of more than 5 % is indicated. The classifications of nuclear size (the mean value of respective classification) for the controls (without the test compounds) were as follows. For CHL cells, most nuclear sizes were approximately 60 to 150 μm^2^, and for L5178Y cells, most nuclear sizes were approximately 50 to 100 μm^2^. Hence, NS (%) was defined as follows: cells with a nuclear size of more than 160 μm^2^ for the CHL cell system and with a nuclear size of more than 110 μm^2^ for the L5178Y cell system were classified as (or exhibiting) NS (%).

### CAs and NS for CHL cells

**Fig. 2:**
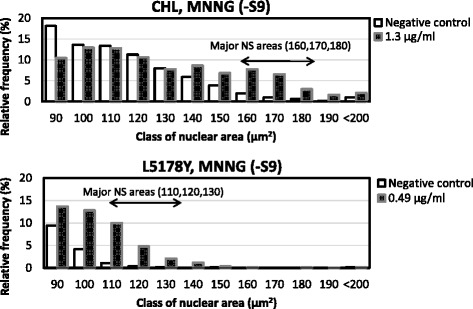
Major nuclear swelling areas for CHL and L5178Y cells. Histogram of cell nuclear areas for clastogens (e.g., MNNG)

NS appeared mainly in the range 160–180 μm^2^; the sum of the relative frequencies (average + SD) of three classes for the negative controls was less than 5 %, and values greater than 5 % were selected to be significant (Fig. 2). Assuming that the nucleus is a sphere, the nuclear volume is proportional to the square root of the cell cross-sectional area raised to the third power; this volume ratio for cell nuclei with NS (%) was 1.1- to 1.3-fold higher than that for the cell nuclei of the negative controls. The occurrence of NS compared to the negative controls occurred in a dose-dependent manner, and NS (%) was strongly correlated with CAs (%) caused by different clastogens (Table 2). However, in detail, MMS (6.3 μg/ml and 13 μg/ml) and MMNG (0.31 μg/ml) did not cause NS, although both clastogens caused CAs. On the contrary, DMBA (2.0 μg/ml) did not cause CAs but caused NS. Regardless, there were strong correlations in this system. For example, the minimum r^2^ of MNNG in this study was 0.84, while MMC and CP had higher r^2^ values of greater than 0.90.

**Table 2:**
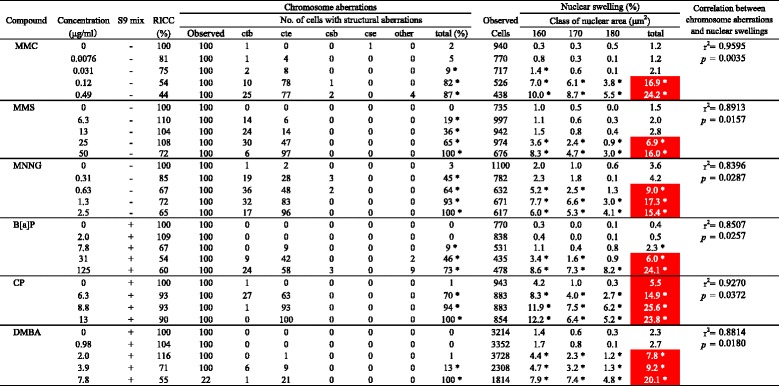
Clastogens induced chromosome aberrations and nuclear swelling in CHL cells

*RICC* relative increase in cell counts

ctb: chromatid break, cte: chromatid exchange, csb: chromosome break, cse: chromosome exchange, others: fragmentation etc

class 160: >150 up to 160 μm^2^, class 170: >160 up to 170 μm^2^, class 180: >170 up to 180 μm^2^

*Asterisks indicate statistically higher than controls (*p < 0.05; Fisher’s exact test or Chi-squared test), White letters indicate clearly higher than controls (NS > 5 %)

### NS of L5178Y cells

**Fig. 3:**
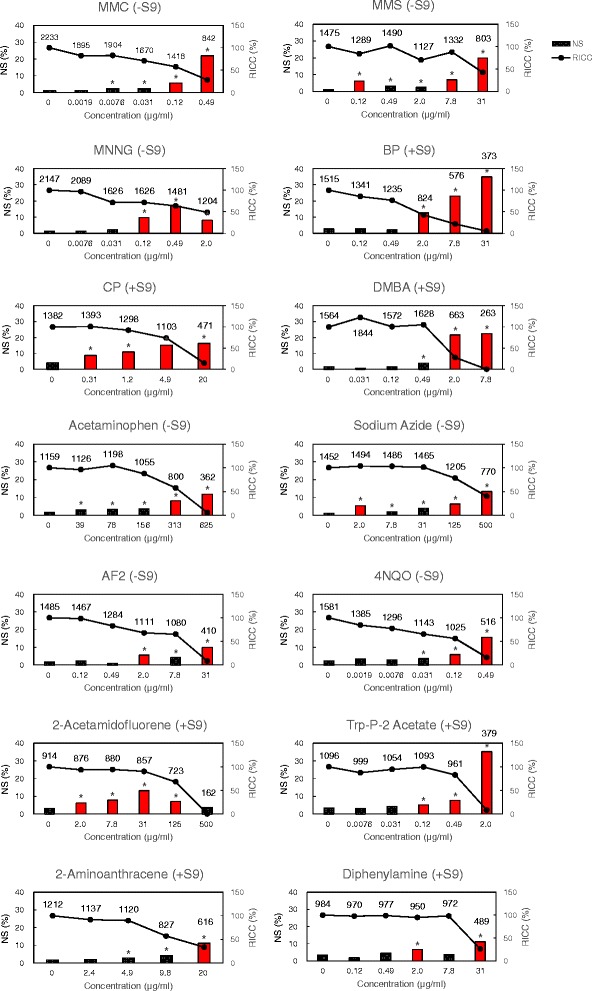
Clastogens induced nuclear swelling in L5178Y cells. NS = nuclear swelling (relative frequency of classes with swelling areas), RICC = relative increase in cell counts. (−S9) = without metabolic activation, (+S9) = with metabolic activation. The label values in the graph are the observed number of cells. Asterisks (*) indicate significant differences compared to controls (*p < 0.05; Fisher’s exact test or Chi-squared test). Nuclear swelling in the red bar graphs is 5 % or more

**Fig. 4:**
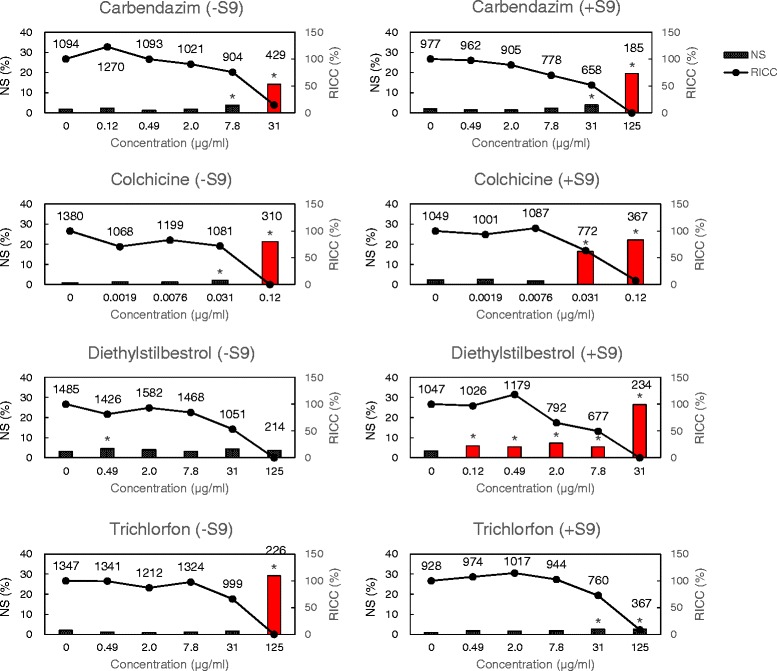
Aneugens induced nuclear swelling in L5178Y cells. NS = nuclear swelling (relative frequency of classes with swelling areas), RICC = relative increase in cell counts. (−S9) = without metabolic activation, (+S9) = with metabolic activation. The label values in the graph are the observed number of cells. Asterisks (*) indicate significant differences compared to controls (*p < 0.05; Fisher’s exact test or Chi-squared test). Nuclear swelling in the red bar graphs is 5 % or more

**Fig. 5:**
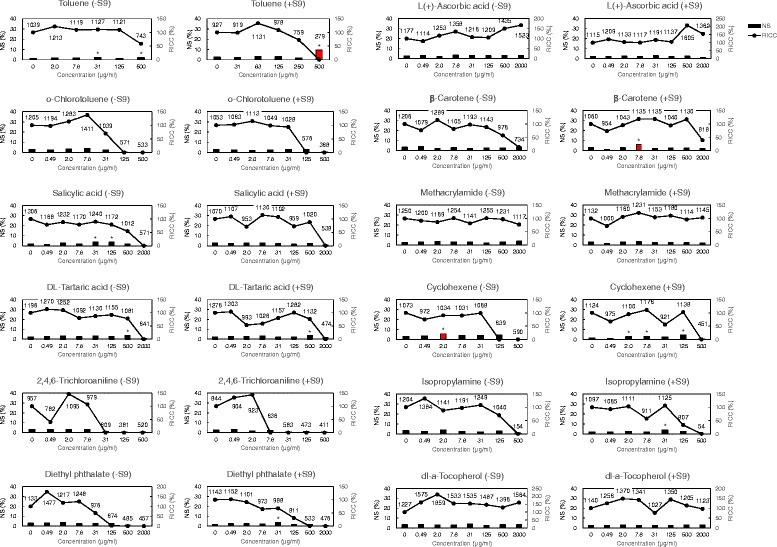
Non-mutagens induced nuclear swelling in L5178Y cells. NS = nuclear swelling (relative frequency of classes with swelling areas), RICC = relative increase in cell counts. (−S9) = without metabolic activation, (+S9) = with metabolic activation. The label values in the graph are the observed number of cells. Asterisks (*) indicate significant differences compared to controls (*p < 0.05; Fisher’s exact test or Chi-squared test). Nuclear swelling in the red bar graphs is 5 % or more

**Fig. 6:**
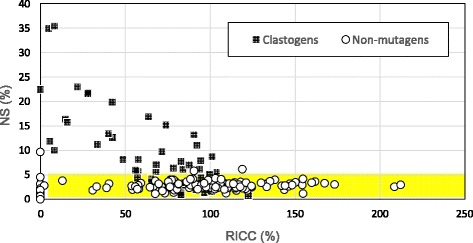
Plots of nuclear swelling vs. RICC in L5178Y cells. NS = nuclear swelling, RICC = relative increase in cell counts

For L5178Y cells, NS appeared in the nuclear size range of 110 – 130 μm^2^ (Fig. 2), and most of the clastogens caused NS in a dose-dependent manner. However, MNNG and 2-acetamidofluorene showed different behaviors, i.e., NS (%) first increased and then decreased after reaching its maximum value, following a bell-shaped curve (Fig. 3). The NS values following aneugen exposure were less than those following clastogen exposure. However, NS (%) values following colchicine and diethylstilbestrol (DES) exposure with metabolic activation were similar to those following clastogen exposures. Moreover, NS (%) was significantly higher at high cytotoxicity levels (Fig. 4). For the non-mutagen test compounds, NS (%) appeared at high cytotoxicity levels, and the values were statistically significant (Fig. 5). Diphenylamine with metabolic activation caused NS at a concentration of 2.0 μg/ml with a frequency of more than 5 %. The relation between RICC (%) and NS (%) indicates that the clastogen more strongly induces NS (%) than the aneugens and non-mutagens (Fig. 6).

## Discussion

In the CHL cell tests using six types of clastogens, each compound showed a high correlation between CAs (%) and NS (%). Therefore, NS (%), which does not depend on the abnormal enhancement of DNA synthesis, was due to the structural CAs in the interphase state. Normal individual chromosomes in interphase cell nuclei have a highly discrete compartment called chromosome territories [25]. Chromosomes damaged by radiation move into these chromosome territories [26]. Since clastogens also damage the structure of chromosomes, consequently, the chromosome territories might be distorted or destroyed. As a result, differences in the strain and stress of chromosome positioning will cause NS in interphase. Some of the differences between NS (%) and CAs (%) may be due to other factors contributing to NS. Since there are various mechanisms for chromosome repair functions, the different characteristics of chemicals might affect the degree of NS (%).

If NS reflects structural CAs, then a larger number of chromosomes in a cell may lead to a greater degree of NS, provided that each chromosome has same basic volume irrespective of the cell nuclear species. For example, the number of chromosomes in CHL cells is 25, while the number in L5178Y cells is 40, and thus the degree of NS in L5178Y cells is expected to be greater than that in CHL cells. However, the experimental results of swelling showed that both cells had similar cross-sections of approximately 30 μm^2^, i.e., both cells showed nearly the same volumetric increase. These results do not support our hypothesis. However, the results suggest that a certain amount of space is required to repair structural chromosome aberrations in the nucleus.

In the L5178Y cell tests, the appearance of NS (%) increased in a dose-dependent manner for almost clastogens tested. However, MNNG and 2-acetamidofluorene showed bell-shaped dose–response curves, and it was concluded that the cytotoxicity of these compounds was a more important factor than NS at high doses. In other words, the appearance of NS is due to factors other than cell death.

AF2 is a typical positive control. On the other hand, one study reported that diphenylamine caused micronuclei formation in human lymphocytes [27], and it is considered a weak clastogen [20]. These compounds caused similar degrees of NS (%).

Colchicine causes NS, and it also causes structural and numerical CAs [28]. Thus, these characteristics are consistent with our experimental results in that colchicine caused higher NS (%) values than the other aneugens. DES with metabolic activation caused NS (%) at more than 5 % relative frequency. There are reports that DES causes sister-chromatid exchanges and structural CAs in vitro in rodent cell lines [29, 30], and it is expected that DES will also cause NS. NS (%) values following exposure to non-mutagens were greater than 5 % at dosages of 500 μg/ml (+S9) for toluene, 7.8 μg/ml (+S9) for β-carotene, and 2.0 μg/ml (+S9) for cyclohexene. NS (%) caused by toluene at high cytotoxicity levels is due to the same phenomenon that caused CAs at high cytotoxicity levels. The non-mutagens tested did not produce clearly defined NS (%) in the present study. These results strongly suggest that NS associated with genotoxicity is caused by structural aberrations in the nucleus during interphase of the cell cycle.

NS during interphase (S, G2) is different from CAs that occur during the mitotic phase, as the cell has passed the G2/M checkpoint, but NS can be used to detect the effect of clastogens. The NS assay is a new method for assessing genotoxicity. This assay can also be applied in conjunction with other tests because it requires an extremely small amount of sample, and the assay can be automated to reduce the amount of time required.

## Conclusions

In a short-term CA test using the fibroblast CHL cell line, the appearance of NS (%) increased in a dose-dependent manner after exposure to six types of clastogens (MMC, MMS, MNNG, BP, CP, and DMBA), and a strong correlation was found between NS (%) and CAs (%) at each dosage. Therefore, we hypothesized that clastogens cause NS in cultured mammalian cells, since the mouse lymphoma L5178Y cell line is known to have a similar sensitivity to clastogens. Thus, we measured NS (%) for 14 compounds (clastogens) that are known to induce structural CAs, 4 aneugens, and 12 non-mutagenes. Almost all clastogens caused NS (%) of more than 5 %, which increased in a dose-dependent manner. Among the aneugens, colchicine, and diethylstilbestrol caused the same level of NS (%) as the clastogens, while carbendazim and trichlorfon caused a similar level of NS (%) as the clastogens only at higher levels of cytotoxicity. Almost all the non-mutagens caused less than 5 % NS. These results strongly suggest that NS is mainly caused by structural aberrations in the nucleus during interphase of the cell cycle.
